# Genomic Analysis of Putative Virulence Factors Affecting Cytotoxicity of *Cronobacter*

**DOI:** 10.3389/fmicb.2019.03104

**Published:** 2020-02-07

**Authors:** Jinghua Cui, Jinrui Hu, Xiaoli Du, Chao Yan, Guanhua Xue, Shaoli Li, Zhigang Cui, Hua Huang, Jing Yuan

**Affiliations:** ^1^Department of Bacteriology, Capital Institute of Pediatrics, Beijing, China; ^2^State Key Laboratory of Infectious Disease Prevention and Control, Collaborative Innovation Center for Diagnosis and Treatment of Infectious Disease, National Institute for Communicable Disease Control and Prevention, Chinese Center for Disease Control and Prevention, Beijing, China; ^3^Beijing Products Quality Supervision and Inspection Institute, Beijing, China

**Keywords:** *Cronobacter* spp., cytotoxicity, hemolysin, fimbria (pilus), species specificity

## Abstract

*Cronobacter* spp. can cause systemic infections, such as meningitis, sepsis, and necrotizing enterocolitis, in immunocompromised patients, especially neonates. Although some virulence factors have been reported previously, the pathogenesis of *Cronobacter* remains unclear. In this study, we compared genome sequences from different *Cronobacter* species, sequence types, and sources, with the virulence genes in the virulence factor database. The results showed that *Cronobacter* has species specificity for these virulence genes. Additionally, two gene clusters, including *sfp* encoding fimbriae and *hly* encoding hemolysin, were discovered. Through cell adhesion, cytotoxicity, and hemolysis assays, we found that the isolates possessing the two gene clusters had higher cytotoxicity and stronger hemolysis capacity than those of other isolates in this study. Moreover, analysis of type VI secretion system (T6SS) cluster and putative fimbria gene clusters of *Cronobacter* revealed that T6SS have species specificity and isolates with high cytotoxicity possessed more complete T6SS cluster construction than that of the rest. In conclusion, the two novel gene clusters and T6SS cluster were involved in the mechanism underlying the cytotoxicity of *Cronobacter*.

## Introduction

*Cronobacter* spp. can cause systemic infections in immunocompromised patients, especially neonatal meningitis, sepsis, and necrotizing enterocolitis in neonates. In 2018, the journal *Emerging Infectious Disease* published four reports on *Cronobacter*, which showed new research topic focusing on *Cronobacter* (Zeng et al., 2015; Chaves et al., 2018; McMullan et al., 2018; Morato-Rodríguez et al., 2018). To date, seven different species of *Cronobacter* have been identified, namely *Cronobacter sakazakii*, *Cronobacter malonaticus*, *Cronobacter turicensis*, *Cronobacter muytjensii*, *Cronobacter dublinensis*, *Cronobacter universalis*, and *Cronobacter condimenti* (Joseph et al., 2012b). These bacteria were observed to adhere to different epithelial cell lines and even persist in macrophage cells (Mange et al., 2006; Townsend et al., 2008). An outer membrane protein called OmpA is essential in the adhesion process of *C. sakazakii* (Singamsetty et al., 2008; Mittal et al., 2009), and the genes *ompX* and *inv* work synergistically with *ompA* contributing the virulence (Kim et al., 2010; Chandrapala et al., 2014). Enterotoxin production by the bacteria was initially evaluated by the suckling mouse assay, and some strains were found to possess the capacity to produce enterotoxins. Moreover, these bacteria have been found to cause cytotoxicity in CHO, Vero, and Y-1 cells (Pagotto et al., 2003). However, after the study conducted by Pagotto et al. (2003), no study analyzing the cytotoxicity of *Cronobacter* was published, until 2017, when Singh et al. (2017) reported the cytotoxicity of *C. sakazakii* N81 in Caco-2 cells. Cytotoxicity is an important virulence factor for bacteria, and toxins and exoenzymes play an important role in bacterial cytotoxicity.

Hemolysins are cytotoxic proteins that target cell membranes, and their mechanisms of damaging membrane integrity can be classified into the following three major groups: enzymatic activity, pore-forming cytolysin, or surfactant (Leclercq et al., 2016). Cytotoxicity induced by *Streptococcus agalactiae*, also known as group B streptococcus, was mediated entirely by the bacterial β-hemolysin/cytolysin (Leclercq et al., 2016). In *Staphylococcus aureus*, α-hemolysin cytotoxic to HL60 human promyelocytic leukemia cells was purified (Tsuiji et al., 2019). Hemolysin in *Vibrio vulnificus* exhibits powerful hemolytic and cytolytic activities and contributes to bacterial invasion from the intestine to the blood stream (Elgaml and Miyoshi, 2017). Although it is known that a gene encoding hemolysin exists in *Cronobacter* (Cruz et al., 2011; Singh et al., 2017), whether this hemolysin is active and associated with cytotoxicity, has not yet been elucidated.

In addition, secretion system and fimbriae also assist the cytotoxicity of bacteria. Type VI secretion system (T6SS) genomic island in *Citrobacter freundii* strain CF74 is involved in the adherence to host cells and induces cytotoxicity in host cells (Liu et al., 2015). The genome construction of T6SS in *Cronobacter* has been revealed previously (Wang et al., 2018), but further studies evaluating its cytotoxicity are required. In *Pseudomonas aeruginosa*, type IV pili are required for promoting close contact between bacteria and the host cell so that exolysin (ExlA) can exert its cytotoxic activity. The interaction of ExlA with membranes results in pore formation, followed by lactic dehydrogenase (LDH) release, and eventually death of infected eukaryotic cells (Basso et al., 2017). *Proteus mirabilis* MR/P fimbriae and flagella mediate genotoxic and cytotoxic effects on eukaryotic cells, at least *in vitro* (Scavone et al., 2015). Purified cbl pili were found to directly induce cytotoxicity in *Burkholderia cenocepacia* strain A549 and activate cysteine proteinases involved in apoptosis (Cheung et al., 2007). Although 10 putative fimbria gene clusters were identified in *Cronobacter*, these genes were not present or functional in any *Cronobacter* species (Joseph et al., 2012a). For example, curli fimbriae in *Cronobacter* were reported to be involved in biofilm formation and cell–cell aggregation (Hu, 2018), whereas *C. sakazakii* and *C. muytjensii* strains were devoid of curli fimbriae genes.

Virulence factor database (VFDB^1^) is an integrated and comprehensive online resource for curating information about virulence factors of bacterial pathogens. In the present study, we compared genome sequences of *Cronobacter* from different species, sequence types (STs), and sources with the virulence genes in VFDB, to determine species specificity and elucidate the association between the virulence-related genes and phenotype.

## Materials and Methods

### Strain Isolation

The stains of *Cronobacter* in this study were isolated from food, water, spice, anus swab, blood, and cerebrospinal fluid (CSF) supplied by 12 province Centers for Disease Control and Prevention of China. The study about clinical strains isolated from blood and CSF has been published (Cui et al., 2017), and ethics approval had been submitted to the Journal. This research was approved by Ethics Committee of National Institute for Communicable Disease Control and Prevention. Thirty-one strains included 15 *C. sakazakii*, 7 *C. malonaticus*, 5 *C. dublinensis*, 2 *C. turicensis*, and 2 *C. muytjensii*. The detailed information is listed in Table 1. The strain *Escherichia coli* HB101 was as negative control. For cell assays, bacterial cultures were prepared by inoculating tryptone soya broth (TSB) medium (Oxiod, United Kingdom) with single colonies grown on tryptone soya agar plates and incubating them aerobically for 16–18 h at 37°C. For logarithmic subcultures, the cultures were diluted 1:100 into fresh TSB and incubated for 4 h at 37°C with shaking (200 rpm). For the growth phase experiments, subcultures were obtained as described above except that the cultures were incubated up to 24 h.

**TABLE 1:** The information of *Cronobacter* isolates in this study.

Strain no.	Species	Source	Isolation time	ST	CC	Crispr 1 no.	Genome accession no.
AH02	*C. sakazakii*	Food	2012	21	21	2	RPAZ00000000
CQ04	*C. sakazakii*	Stool	2012	13	13	1	RPBC00000000
HA03	*C. sakazakii*	Food	2012	209		1	RPBD00000000
HB04	*C. sakazakii*	CSF	2014	83	83	1	LGRL00000000
HE35	*C. sakazakii*	Food	2012	64	64	1	RPBE00000000
LN01	*C. sakazakii*	Food	2006	23	23	1	RPBF00000000
LN02	*C. sakazakii*	Food	2006	235	4	1	RPBG00000000
SC16	*C. sakazakii*	Food	2011	4	4	1	RPBJ00000000
SC25	*C. sakazakii*	Food	2010	1	1	1	RPBK00000000
SC26	*C. sakazakii*	Stool	2015	42		1	RPBI00000000
SD04	*C. sakazakii*	Spice	2010	144	40	1	RPBL00000000
SD19	*C. sakazakii*	Water	2010	151		1	RPBP00000000
SD45	*C. sakazakii*	Stool	2011	40	40	1	RPCB00000000
SD47	*C. sakazakii*	Food	2011	155	155	1	RPBZ00000000
XJ02	*C. sakazakii*	Food	2012	12		1	RPBV00000000
BJ15	*C. malonaticus*	Stool	2013	7	7	1	RPBB00000000
HB03	*C. malonaticus*	Blood	2014	60		1	LGRM00000000
SC01	*C. malonaticus*	Food	2010	201	7	1	RPBH00000000
SD11	*C. malonaticus*	Water	2010	169		2	RPBN00000000
SD16	*C. malonaticus*	Water	2010	7	7	1	RPBO00000000
SD26	*C. malonaticus*	Water	2010	159	7	1	RPBR00000000
SD43	*C. malonaticus*	Water	2010	160	62	1	RPCA00000000
BJ09	*C. dublinensis*	Food	2007	239		1	RPBA00000000
SD05	*C. dublinensis*	Plant	2010	167		2	RPBM00000000
SD28	*C. dublinensis*	Water	2010	173		1	RPBS00000000
SD69	*C. dublinensis*	Spice	2016	43		0	RPBY00000000
SX10	*C. dublinensis*	Food	2016	80	80	5	RPBW00000000
SD21	*C. turicensis*	Water	2010	171	147	1	RPBQ00000000
SH11	*C. turicensis*	Stool	2013	309		1	RPBX00000000
SD83	*C. muytjensii*	Spice	2016	489		0	RPBU00000000
SD92	*C. muytjensii*	Spice	2016	493		1	RPBT00000000

### DNA Preparation and Sequencing

The genomes of *Cronobacter* strains were sequenced by BGI Tech Solutions Co. Ltd., Beijing, China. Sequences were generated with an Illumina HiSeq2000 (Illumina Inc., San Diego, CA, United States). Quality trimming of 150-nucleotide (nt) paired-end reads, produced from a 500-bp genomic library, and subsequent assembly were performed using SOAP *de novo* v1.05 (Li et al., 2010). Raw data were processed in four steps involving the removal of reads with ambiguous bases (1–90 bp), 20 bp of low quality reads (≤Q20), adapter contamination, and duplicated reads.

### Bioinformatics Analysis

DNA sequences were obtained in this study and were compared with reference sequences in NCBI database, using BLAST software 2.2.22 of tblastx program with amino acid sequence. The sequences were then screened by identity value (>50%), and heat maps constructed with coverage value. The heat maps were prepared with pheatmap package in R 3.5.1 and cluster analysis performed with ward.D method. Sequences in all the strains were compared with those of the virulence genes in the virulence factor database (VFDB, see text footnote 1), which is an integrated and comprehensive online resource for curating information about virulence factors of bacterial pathogens. The respective virulence genes from five species of *Cronobacter* were analyzed with Venn diagram^2^.

### Nucleotide Sequence Accession Numbers

This Whole Genome Shotgun project has been deposited at GenBank under the BioProject PRJNA287482 and PRJNA498360, with accession numbers LGRM00000000, LGRL00000000, RPAZ00000000, RPBA00000000-RPBZ00000000, RPCA00000000, and RPCB00000000.

### Growth Curve

Isolates were incubated overnight and then transferred into 20 ml of Dulbecco’s modified Eagle’s medium (DMEM) at ratio of 1:100. The isolates were then grown at 37°C with shaking at 180 rpm/min. OD 600 was measured every hour for each isolate.

### HEp-2 Adhesion and Cytotoxicity Assay

The human epidermoid laryngocarcinoma (HEp-2) cell line was obtained from the National Institute for Viral Disease Control and Prevention, China CDC. The cells were maintained in flasks containing DMEM (Gibco, United States) supplemented with 10% fetal bovine serum (Gibco, United States), at 37°C in 5% CO_2_. A mixture with a multiplicity of infection (number of bacteria per number of mammalian cells) of 100:1 was added to the HEp-2 monolayers in a 1-ml culture medium. Bacteria were allowed to adhere for 3 h at 37°C in 5% CO_2_. Non-adherent cells were removed by washing three times with phosphate-buffered saline (Gibco, United States), and adherent cells were collected using cell scraper and were then transferred to 1.5-ml tubes with 1 ml of phosphate-buffered saline. Using the plate dilution method, the bacterial cells were measured. The interaction rate was expressed as (interaction cell number)/(inoculated cell number) × 100%. *E. coli* HB101 and enteropathogenic *E. coli* 2348/69 were used as negative and positive controls for adhesion assay, respectively.

For cell cytotoxicity assay, bacteria were incubated in cells with a multiplicity of infection of 100:1 for 8 h at 37°C in 5% CO_2_. The mixture was collected and centrifuged at 4,000 rpm for 5 min. The supernatant was used to detect the release of LDH using the CytoTox96 kit (Promega, United States) according to the manufacturer’s instructions. Lysed cells and cells with *E. coli* strain HB101 were used as positive and negative controls, respectively. The relative amount of cytotoxicity was expressed as (experimental release − spontaneous release)/(maximum release − spontaneous release) × 100%, in which the spontaneous release was the amount of LDH activity in the supernatant of uninfected cells and the maximum release was that when cells were lysed with the lysis buffer provided by the manufacturer. All experiments were performed thrice.

### Hemolysis Assay

Hemolytic activity was evaluated using Columbia Blood Agar Plate (Oxiod, United Kingdom) containing 5% of erythrocytes from sheep. Bacteria were grown in 1 ml TSB, at 37°C with shaking at 180 rpm/min overnight and centrifuged at 4,000 rpm for 5 min. The supernatant was discarded, and the pellet was resuspended with residual supernatant. Five microliter of the above mixture was dropped onto blood agar plates and incubated at 37°C for 24 h. The isolates were evaluated according to the cleared zone produced.

### Statistical Analysis

Data were analyzed using the SPSS 20.0 statistical software packages. All values are presented as the means ± standard deviation (mean ± SD). The level of statistical significance was determined using a *t-*test. The statistical significance was set at *P* < 0.05.

## Results

### Genome Analysis of Virulence Genes for Different Species of *Cronobacter* Isolates

**FIGURE 1:**
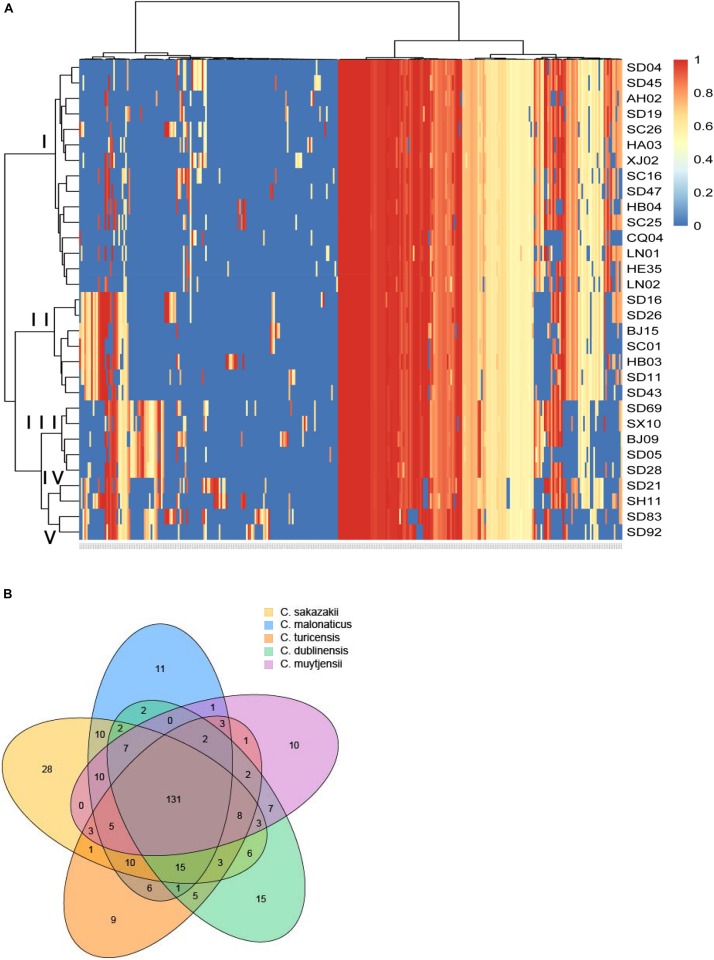
Genome analysis of virulence genes for different species of *Cronobacter* isolates. **(A)** Hierarchically clustered heatmap of the virulence genes distribution of 31 *Cronobacter* isolates. The heatmap plot depicts the sequences coverage value of virulence genes (horizontal axis clustering) within each isolate (variables clustering on the vertical axis). The sequences coverage value of virulence genes are indicated by color intensity with the legend indicated at the right top of the figure. **(B)** Sequence diversity and functional enrichment across clustered genes. Virulence genes from each species were clustered using OrthoFinder v.0.7.1. This yielded 319 genes, shown here using draw quad. venn from the VennDiagram package of Rstudio v.3.0.2. Ovals with no cross indicate singleton genes possessed by every species of *Cronobacter*, and ovals with cross represent clusters of genes that have orthologs and paralogs for different species of *Cronobacter*.

In this study, a total of 319 virulence genes were analyzed after comparison with the virulence genes of VFDB (Supplementary Table S1). Using the ward.D method, we found that the distribution of genes in the same species of all the isolates in this study was similar and clustered. As shown in Figure 1A, all the virulence genes of the isolates were clustered in five groups: group I was *C. sakazakii*, group II was *C. malonaticus*, group III was *C. dublinensis*, group IV was *C. turicensis*, and group V was *C. muytjensii*. Therefore, *Cronobacter* has species specificity for these virulence genes. However, no difference in gene content seemed to contribute to the differences in ST or source. Among these virulence genes, 131 genes were collectively present in the five species of *Cronobacter*. Furthermore, there were 28 virulence genes in *C. sakazakii*, 11 in *C. malonaticus*, 15 in *C. dublinensis*, 9 in *C. turicensis*, and 10 in *C. muytjensii* possessed by different species (Figure 1B).

### Analysis of Virulence Genes Related to Adhesion, Toxins, and Secretion System

**FIGURE 2:**
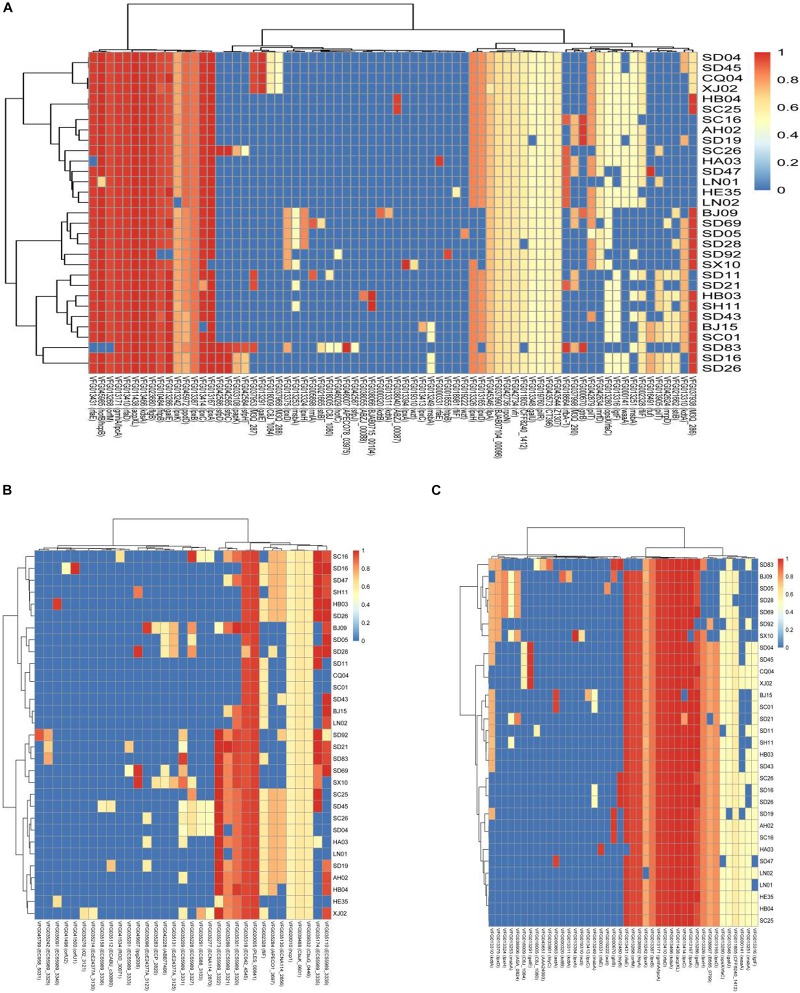
Hierarchically clustered heatmap of the genes encoding adhesion **(A)**, toxin **(B)**, and secretion system **(C)** for 31 *Cronobacter* isolates in this study. The heatmap plot depicts the sequences coverage value of genes encoding adhesion, toxin, and secretion system (horizontal axis) with in each isolate (vertical axis).

We selected the genes encoding virulence factors related to adhesion, toxins, and secretion system and compared them with the virulence genes in VFDB. Most of the isolates in this study harbored the genes *acpXL*, *galE*, *gmhA*/*lpcA*, *lpxA*, *lpxC*, *kdsA*, *rfaD*, *rfaE*, and *orfM*, which encode both adhesion and toxins (Figures 2A,B). The genes *htpB*, *flaB*, and *hofB/hcpB* associated with adhesion existed in most of the isolates analyzed in this study (Figure 2A), and the gene *hlyA* encoding hemolysin was detected in all the isolates except the isolate SD 83 (Figure 2B). Notably, some particular genes belonged only to a few specific isolates. A gene cluster consisting of the genes *sfpC*, *sfpD*, and *sfpH* was only found in the isolates *C. sakazakii* SC26, *C. malonaticus* SD16, *C. malonaticus* SD26, and *C. muytjensii* SD83, and was similar to the genes encoding bundle-forming pilus protein in sorbitol-fermenting enterohaemorrhagic *E. coli* O157:H (−) (Figure 2A). Interestingly, the four above-mentioned isolates harbored the gene *hlyB* encoding an inner membrane protein with a cytoplasmic ATP-binding cassette domain, which can pump out HlyA protein (Benabdelhak et al., 2003). All the isolates harbored two genes (PLES_00841 and EC042_4545) encoding a hypothetical protein belonging to the T6SS (Figure 2C). Some isolates possessed genes such as EC55989_3320, EC55989_3321, EC55989_3335, and EC55989_3339, which also encoded hypothetical proteins belonging to the T6SS.

### HEp-2 Cell Adhesion and Cytotoxicity of *Cronobacter* Isolates

**FIGURE 3:**
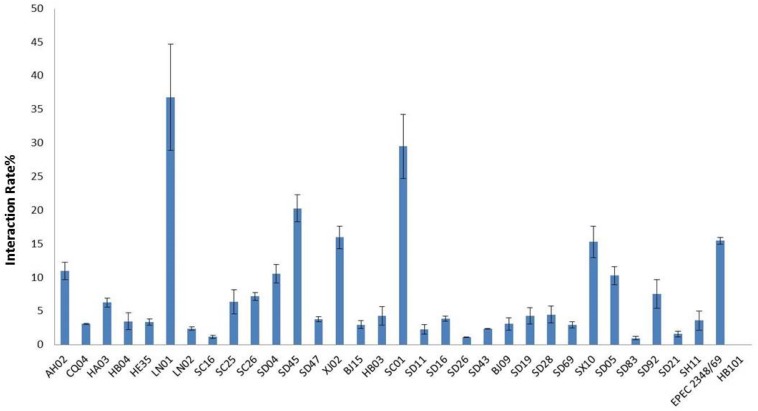
*Cronobacter* attached to the Hep-2 epithelial cell surface was enumerated after 3 h by subtracting the number of bacteria recovered following. Results are presented as the percentage of the inoculum associated. Data are means ± standard errors of two independent experiments performed in triplicate. *E. coli* HB101 and enteropathogenic *E. coli* (EPEC) 2348/69 were used as negative and positive controls, respectively.

**FIGURE 4:**
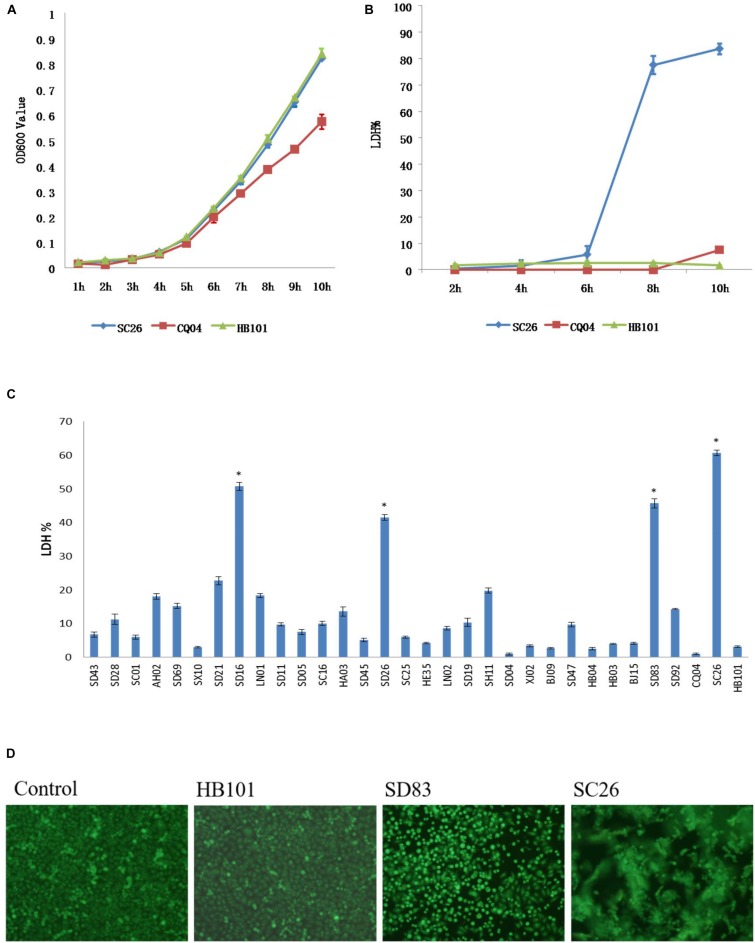
Lactate dehydrogenase (LDH) released by HEp-2 cells after growth with *Cronobacter* and growth curve of these isolates in Dulbecco’s modified Eagle’s medium (DMEM) media. **(A)** LDH released by HEp-2 cells after growth with isolates *C. sakazakii* SC 26, *C. sakazakii* CQ04, and *E. coli* HB101 at 2, 4, 6, 8, and 10 h. **(B)** The growth curve of isolates *C. sakazakii* SC 26, *C. sakazakii* CQ04, and *E. coli* HB101 in DMEM at 1-h intervals within 10 h. **(C)** LDH released by HEp-2 cells after growth with 31 *Cronobacter* isolates. Negative control was isolate *E. coli* HB101. ^∗^LDH value of four isolates that could cause cell rounding and death were much higher than other isolates (*P* < 0.05). **(D)** Morphological changes of HEp-2 cells after growth with *Cronobacter* isolates. Cell rounding, detachment, and death can be seen after 8 h of exposure to *C. muytjensii* SD83 and *C*. *sakazakii* SC26. *E. coli* HB101 was used as negative control.

All isolates in this study were tested for cell adhesion and cytotoxicity to cultured HEp-2 cells. The average interaction rate of all the isolates was 7.5%, and eight of those isolates were above an average value (Figure 3). The interaction rates of the isolates from different sources were compared, and the results showed that there were no significant differences among them (*P* > 0.05). The same results were obtained for different STs and species of *Cronobacter*. The four isolates with *sfp* gene cluster did not show more higher adherence capacity than that of other isolates. Therefore, whether *sfp* gene cluster in *Cronobacter* is associated with adhesion warrants further research. Among the isolates, difference in cytotoxicity in HEp-2 cells was determined by measuring the amount of LDH released by HEp-2 cells when cocultured with *Cronobacter*. We first cultured isolates *C. sakazakii* SC26 and *C. sakazakii* CQ04 in DMEM and detected the OD 600 value hourly. The isolates *C. sakazakii* SC26 and *C. sakazakii* CQ04 exhibited a slow growth in the media, and no difference was observed between these isolates including HB101 (Figure 4A). However, the release of LDH was statistically significantly different at 8 h (Figure 4B). We then tested the remaining isolates at 8 h and found only four isolates (*C. sakazakii* SC26, *C. malonaticus* SD16, *C. malonaticus* SD26, and *C. malonaticus* SD83) that could cause cell rounding and death among the 31 strains (Figure 4D). The average LDH released was 14.12%, ranging from 0.95 to 60.64%. Most isolates showed a rather small range of LDH release, from 0.95 to 22.76% with an average of 8.85%, whereas in the four above-mentioned isolates, LDH release ranged between 41.52 and 60.64%, with an average of 49.66%, which was much higher than that of other isolates (*P* < 0.05, Figure 4C). There was no obvious trend in the difference in cytotoxicity between STs and clusters.

### Hemolysis and Gene Cluster Analysis of *Cronobacter* Isolates

**FIGURE 5:**
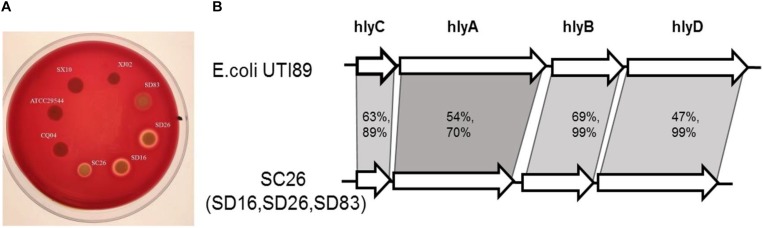
Hemolysis phenotype and gene constrion for *Cronobacter*. **(A)** Different isolates showed different hemolysis capicity on blood agar plate. **(B)** The gene cluster comparision of the four isolates with strong hemolysiscapicity with *E. coli* UTI89.

Hemolysis was tested in all the isolates in this study, and the results showed that most of isolates were negative, except for the isolates *C. sakazakii* SC26, *C. malonaticus* SD16, *C. malonaticus* SD26, and *C. muytjensii* SD83. As shown in Figure 5A, the four isolates formed a hemolysis ring around these isolates. The isolates *C. malonaticus* SD16, *C. malonaticus* SD26, and *C. sakazakii* SC26 showed high hemolysis capacity, followed by *C. muytjensii* SD83. Next, we analyzed the flanking region sequences of *hlyB* and found a gene cluster, comprising the genes *hlyC*, *hlyA*, *hlyB*, and *hlyD*, similar to *E. coli* UTI89 (Figure 5B). By comparing the amino acid sequences of *E. coli* UTI89, we found that the coverage of *hlyB* and *hlyD* was 99%, *hlyC* was 89%, and *hlyA* was 70%, and the identity of *hlyB*, *hlyC*, *hlyA*, and *hlyD*, was 69, 63, 54, and 47%, respectively. The length of whole gene cluster in *E. coli* UTI89 (7,380 bp) was longer than that in *Cronobacter* (6,771 bp); therefore, the function of the gene cluster in *Cronobacter* needs to be elucidated.

### Known Virulence Genes of *Cronobacter* Isolates in Connection With Cytotoxicity

**FIGURE 6:**
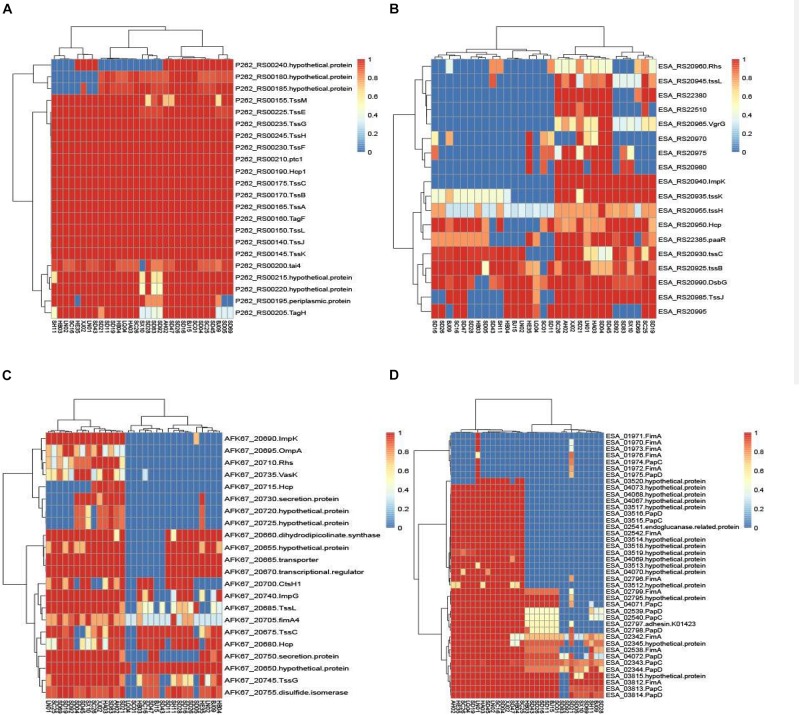
The heatmap was generated based on 31 *Cronobacter* isolates for genes encoding T6SS and fimbriae. The heatmap plot depicts the sequences coverage value of genes encoding T6SS and fimbriae (vertical axis) with in each isolate (horizontal axis). **(A)** T6SS cluster in chromosome of *C. malonaticus* NC_023032 was used as the reference sequences; **(B)** sequences of T6SS cluster in *C. sakazakii* ATCC BAA-894 pESA3 was used as the reference; **(C)** sequences of T6SS cluster in *C. dublinensis* LMG 23823 pCDU1 was used as references; and **(D)** 10 putative fimbriae gene clusters were as reference sequences.

The chromosomal T6SS clusters of different species were downloaded from GeneBank and compared (data not shown). Since two isolates (*C. malonaticus* SD16 and *C. malonaticus* SD26) belonged to *C. malonaticus*, the T6SS cluster in chromosome of *C. malonaticus* NC_023032 was used as the reference sequence in this study (Figure 6A). Since there were reports of T6SS cluster in plasmid, the sequences of T6SS cluster in *C. sakazakii* ATCC BAA-894 pESA3 (Figure 6B) and *C. dublinensis* LMG 23823 pCDU1 (Figure 6C) were used as references. After comparing T6SS clusters of all the isolates with their reference sequences, we found the four above-mentioned strains to possess more complete T6SS clusters than other isolates. Therefore, the complete content of T6SS clusters might be helpful for the release of toxin or protein enzyme causing cytotoxicity.

Ten putative fimbriae gene clusters, reported by Joseph et al. (2012a), were as reference sequences. Sequences of all the isolates in this study were compared with those of the reference genes, and results showed the genes encoding fimbriae, for different species of *Cronobacter*, to be species specific. As in Figure 6D, 15 *C. sakazakii* isolates were found to be more similar to the reference than other isolates. Except for the isolate *C. sakazakii* LN01, gene cluster named ESA_01970–ESA_01976 of other 14 strains were absent in *C. sakazakii*. For the other species *C. malonaticus*, *C. dublinensis*, *C. turicensis*, and *C. muytjensii*, sequence similarity with the reference was <50%. The seven *C. malonaticus* isolates possessed only two complete gene clusters, namely, ESA_02342–ESA_02345 and ESA_03812–ESA_03815, whereas the isolates belonging to *C. dublinensis*, *C. turicensis*, and *C. muytjensii* had no complete gene cluster encoding fimbriae. These putative fimbrial gene clusters seemed to have no bearing on cytotoxicity, since the four above-mentioned isolates had no common characteristics in terms of these genes.

## Discussion

Although some virulence genes have been reported previously, including two iron acquisition system loci (eitCBAD and iucABCD/iutA), a two-partner secretion system/filamentous hemagglutinin gene (fhaB), a transporter gene (fhaC), associated putative adhesins (FHA) locus, and a T6SS locus, hemolysin III (*hly*), and 10 putative fimbriae (pilus) gene clusters (Cruz et al., 2011; Franco et al., 2011; Joseph et al., 2012a), the genes were found only in the subsets of genomes, and all contributed considerably to the variation in gene content. By comparing the genes of all the isolates in this study with those in VFDB, the notable features of the different species of *Cronobacter* and a new virulence factor were discovered. Cluster analysis was performed with ward.D method on all the virulence genes aligned with that of VFDB, and results revealed the isolates from same species to be clustered together, thereby demonstrating that the isolates of same species possessed similar virulence genes. *Cronobacter* being a highly clonal organism, the genes are coinherited owing to the clonality (restricted genome variation) and not due to function, and the difference in these virulence genes might be caused by evolution. The isolates belonging to the clonal complex, such as *C. malonaticus* SD16, SD26, BJ15, and SC01, were clustered together; however, little difference existed between these virulence genes. The cas protein-coding gene (CRISPR-cas) array profiling has been applied to *Cronobacter* genomes of strains from the same ST. Moreover, it was demonstrated that strains in the same ST are distinguishable according to their spacer arrays (Ogrodzki and Forsythe, 2016). The presence and differentiated activity of CRISPR appear in different *Cronobacter* species (Zeng et al., 2018). The genome sequences of the isolates in this study were submitted to CRISPRCasFinder^3^, and the results showed that most of the isolates, except for *C. dublinensis* SD69 and *C. muytjensii* SD92, possessed Crispr 1 (Table 1). It demonstrated that no significant variation existed between these isolates.

Many new gene clusters encoding virulence factors in these isolates were discovered. Here, the two gene clusters were analyzed especially because they existed in the same isolates and may have some relevance. As shown in Figure 2A, the four isolates (*C. sakazakii* SC26, *C. malonaticus* SD16, *C. malonaticus* SD26, and *C. muytjensii* SD83) harbored a specific gene cluster, which was similar to the *sfp* gene cluster encoding the bundle-forming pilus protein. The *sfp* gene cluster was first reported in a plasmid of sorbitol-fermenting enterohemorrhagic *E. coli* O157:H(−) and mediates mannose-resistant hemagglutination and fimbrial expression (Brunder et al., 2001). The *sfp* cluster included six genes, namely s*fpA*, *sfpH*, *sfpC*, *sfpD*, *sfpJ*, and *sfpG*. Expression of Sfp fimbriae in sorbitol-fermenting *E. coli* O157: NM strain is induced under conditions resembling those at the natural site of infection, and it may contribute to the adherence of the organisms to human intestinal epithelium (Müsken et al., 2008). Although the gene content of *sfp* in *Cronobacter* was not completed, we speculated the gene cluster to be similar to that in enterohemorrhagic *E. coli* O157: H (−), which had the same function. Another important gene cluster encoding hemolysin was found in these four isolates. The sequences of gene *hlyB* and flanking region were compared with the ones of *E. coli* UTI89, following which a new and completed gene cluster, including *hlyA*, *hlyB*, *hlyC*, and *hlyD*, was identified (Figure 5B). However, notably, the gene *hlyA* of the new cluster was different from the gene *hlyA* (VFG038902) that existed in most of the isolates in this study. It has been reported that *hlyA* and *hlyB* are coexpressed in a non-hemolytic *E. coli* strain, and this strain showed enhanced hemolytic activity on blood agar plates. In addition, the synergistic hemolytic activity of HlyA and HlyB has been detected by liquid hemolytic assay. Moreover, the gene (*hlyIII*) encoding type III hemolysin had been reported to be associated with the hemolytic phenotype of *Cronobacter* (Cruz et al., 2011; Singh et al., 2017), although it was different from the gene cluster discovered in this study. Accordingly, we speculated that the new gene cluster encoding hemolysin was involved in the mechanism underlying hemolysis in *Cronobacter*.

To assess the function of the two gene clusters, we performed cell adhesion, cytotoxicity, and hemolysis assays for the isolates in this study. Most of the *Cronobacter* isolates induced the low release of LDH from cells, but only four isolates (*C. sakazakii* SC26, *C. malonaticus* SD16, *C. malonaticus* SD26, and *C. muytjensii* SD83) induced the cell death in a well after their incubation for 8 h, and LDH values were found to be much higher than in other isolates (*P* < 0.05). Simultaneously, the four isolates showed strong hemolysis capacity, while other isolates were negative. These results were consistent with the gene content characteristics discovered by genome sequencing analysis. Therefore, it was speculated that the two gene clusters, including *sfp* and *hly*, play an important role in the pathogenesis of *Cronobacter*.

Secretion systems are the major weapons required for colonization, survival, cytotoxicity, and evasion of the host innate immune system (Bleves et al., 2010). T6SS is a novel and complex multicomponent secretion system, which is often involved in the interaction with eukaryotic hosts, irrespective of it being a pathogenic or a symbiotic relationship (Filloux et al., 2008). *In silico* analysis of pESA3, harbored by *C. sakazakii* ATCC BAA-894, revealed that this T6SS gene cluster consists of 16 open reading frames (Franco et al., 2011). Besides the plasmid, in the chromosome of *C. sakazakii* ATCC12868, the two phylogenetically distinct T6SS gene clusters (T6SS-1 and T6SS-2) were also investigated (Wang et al., 2018). We compared the T6SS cluster of strains with the chromosome of *C. malonaticus* (NC_023032), plasmid pESA3 of *C. sakazakii* ATCC BAA-894, and pCDU1 of *C. dublinensis* subsp. *dublinensis* LMG 23823, and found T6SS cluster to have species specificity and that isolates with high cytotoxicity possessed more complete T6SS cluster construction than the rest. However, there was no obvious difference between the isolates with high cytotoxicity and other isolates. Therefore, although T6SS cluster might be involved in the mechanism underlying the cytotoxicity of *Cronobacter*, it may not be a key element.

Fimbriae (pili) were considered to be another important element for cytotoxicity of bacteria. Few reports have described the fimbrial characteristics of *Cronobacter*, except for the 10 putative fimbrial gene clusters identified by Joseph et al. (2012a). Although an individual gene cluster may be absent (for example, curli fimbriae genes were absent in *C. sakazakii*), most gene clusters were present in the 12 strains analyzed in the study. In the current study, *C. sakazakii* isolates had gene sequences more similar to the reference sequence, and sequences of other species were <50% similar to that of reference gene cluster. Nevertheless, results showed the gene distribution to have species specificity characteristic.

## Conclusion

By comparing with the virulence genes of VFDB, we found that *Cronobacter* has species specificity for these virulence genes. Besides, the two gene clusters, including *sfp* encoding fimbriae and *hly* encoding hemolysin, were analyzed only because they existed in the same isolates (*C. sakazakii* SC26, *C. malonaticus* SD16, *C. malonaticus* SD26, and *C. muytjensii* SD83). Moreover, these four isolates showed higher cytotoxicity and stronger hemolysis capacity than those of other isolates in this study. Therefore, it was considered that the *hly* gene cluster discovered in this study was associated with hemolysis and cytotoxicity of *Cronobacter*. Although the *sfp* gene cluster seemed to not be associated with adhesion of *Cronobacter*, it might mediate cytotoxic effects on cells. The mechanism of cytotoxicity induced by hemolysin and fimbriae in *Cronobacter* should be studied in the future.

## Data Availability Statement

The datasets generated for this study can be found in the Whole Genome Shotgun project at GenBank under the BioProject PRJNA287482 and PRJNA498360, with accession numbers LGRM00000000, LGRL00000000, RPAZ00000000, RPBA00000000-RPBZ00000000, RPCA00000000, and RPCB00000000.

## Ethics Statement

The studies involving human participants were reviewed and approved by the Ethics Committee of National Institute for Communicable Disease Control and Prevention. Written informed consent to participate in this study was provided by the participants’ legal guardian/next of kin.

## Author Contributions

JC, HH, and JY conceived and designed the experiments. JH, XD, GX, and SL performed the experiments. ZC and CY analyzed the data. JC, JH, XD, and ZC prepared the manuscript. All authors read and approved the final manuscript.

## Conflict of Interest

The authors declare that the research was conducted in the absence of any commercial or financial relationships that could be construed as a potential conflict of interest.

## Footnotes

1 http://www.mgc.ac.cn/VFs/

2 http://www.ehbio.com/ImageGP/index.php/Home/Index/VennDiagram.html

3 https://crispr.i2bc.paris-saclay.fr

## Supplementary Material

TABLE S1 The identification and coverage of virulence genes of *Cronobacter* in this study compared with VFDB.
